# Supplementary material for: Differential Tropism in Roots and Shoots of Resistant and Susceptible Cassava (Manihot esculenta Crantz) Infected by Cassava Brown Streak Viruses
Source: Cells. 2021 May 17;10(5):1221. doi: 10.3390/cells10051221 (PMC8156387; doi:10.3390/cells10051221)
Supplement: Supplementary file 1 [file cells-10-01221-s001.zip › cells-1219508-supplementary.pdf]

cells

MDPI

Article

Differential tropism in roots and shoots of resistant and susceptible cassava (Manihot esculenta Crantz) infected by cassava brown streak viruses

Samar Sheat, Paolo Margaria and Stephan Winter*

Supplement Figures

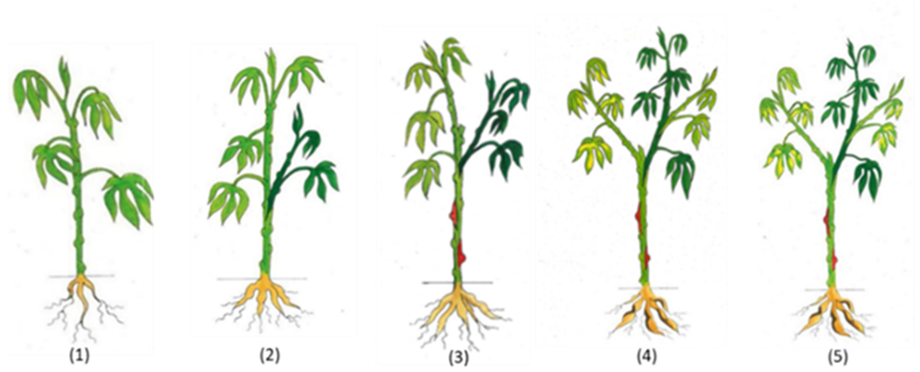

Figure S1. Tracing virus movement in resistant cassava. A scion from a resistant cassava was side grafted onto a rootstock of TMS 96/0304 (1) to establish a chimeric plant (2). Virus infections were introduced by grafting (3). A TMS 96/0304 scion was grafted onto the resistant branch (4) to monitor virus movement (5). Light green, susceptible cassava; dark green, resistant cassava; orange, buds from virus infected plants.

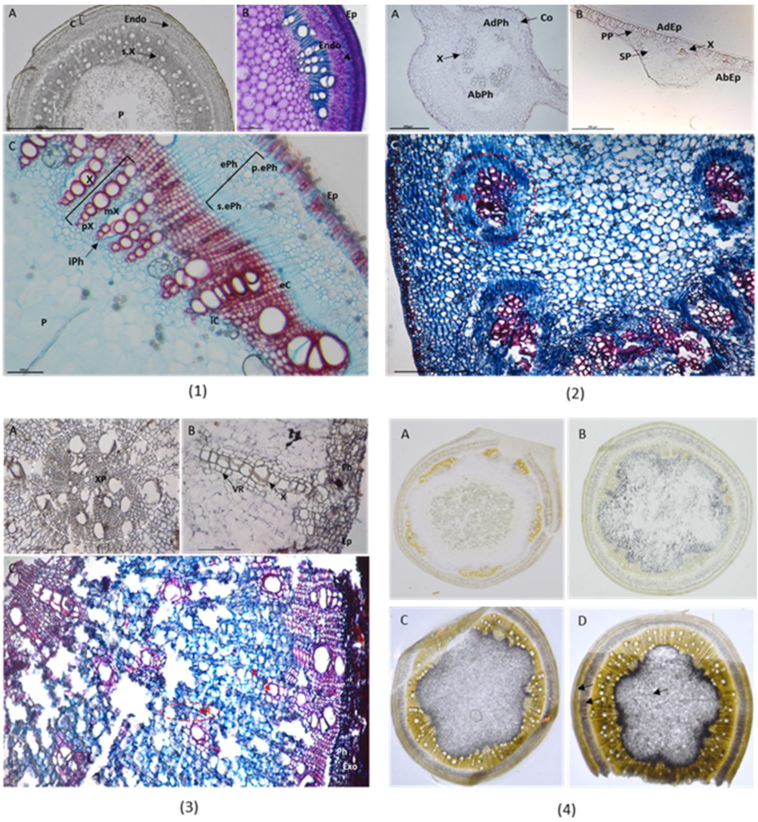

Figure S2. Cassava anatomy. 1- Cross-section of a cassava stem (A). Light microscopy images of a cross-section. (B) TBO staining; lignin, dark blue. (C) FCA staining; lignified cells red, not lignified cells light blue. 2- Cross-section through cassava leaf main vein, midvein, lateral vein and leaf blade tissues. Light microscopy images of the unstained cross-section from cassava (A) midvein tissue and (B) lateral vein. (C) Main vein tissue section stained with Safranin O and Astrablue. Lignin is red, non-lignified tissue blue. VB, vascular bundles, open circle. 3- Cross-section of a cassava root tuber. (A, B) Light microscopy images of the unstained cross-section from cassava tuberous roots. (C) Panorama image from tuberous root section stained with Safranin O and Astrablue. Lignin is red, non-lignified tissue, blue. Xp, xylem poles primary xylem, white open circle; VR, vascular ray parenchyma cells, red open circle.4- Cross-section of cassava stems stained with Lugol solution. Arrows point at containing tissues in the phyllogen, endodermis (starch sheath) and parenchyma cells. (A) Upper stem, (B and C) middle parts of stem and (D) the base of stem. Sections diameter ~ 4 mm. PP, palisade parenchyma; SP, spongy parenchyma; P, parenchyma; AdEp, adaxial epidermis; AbEp, abaxial epidermis; Ep, epidermis; Endo, endodermis; ; Exo, exodermis; Co, collenchyma cells; X, xylem; mX, Meta xylem ; pX, protoxylem; s.X, secondary xylem; C, cortex; AbPh, abaxial phloem; AdPh, adaxial phloem; ePh, external phloem; p.ePh, primary external phloem; s.ePh, secondary external phloem; iPh, inner phloem; Ph, phloem; eC, external cambium; iC, internal cambium.
